# Editorial: Autism spectrum disorder within neurodevelopmental disorders: Catching heterogeneity, specificity, and comorbidity in clinical phenotypes and neurobiological bases

**DOI:** 10.3389/fnins.2022.958650

**Published:** 2022-08-03

**Authors:** Eugenia Conti, Marco Turi, Francesco Craig

**Affiliations:** ^1^Child Neuropsychiatry Unit, ASL Toscana Centro, Empoli, Italy; ^2^Fondazione Stella Maris Mediterraneo Onlus, Matera, Italy; ^3^Department of Cultures, Education and Society, University of Calabria, Cosenza, Italy; ^4^Scientific Institute, IRCCS E. Medea—Unit for Severe Disabilities in Developmental Age and Young Adults, Brindisi, Italy

**Keywords:** heterogeneity, autism spectrum disorder, phenotypes, comorbidity, neurobiological mechanism

Autism spectrum disorder (ASD) refers to a group of complex neurodevelopment disorders (NDDs) characterized by impaired social interaction and communication, and the occurrence of restricted interests and repetitive behaviors (APA, 2013). ASD is characterized by heterogeneity in terms of behavioral expression, onset, treatment-response, and comorbidities, along with heterogeneous genetic and neurobiological underpinnings (Lombardo et al., 2019). Although hinted at in pioneering descriptions by Kanner (1943) and later argued by Wing and Gould (1979), heterogeneity remains poorly defined. Therefore, to understand specific etiologies and inform individualized treatments, it is crucial to move away from the single entity toward addressing “heterogeneity” in ASD. In this frame, it is of utmost importance to keep in mind the “specificity” concept to differentiate ASD from other NDDs and enlarge knowledge about “comorbidity,” too.

Recent state-of-the-art neuroimaging and electrophysiological tools, combined with advanced person-centered analytical and computational approaches, allow our field to start addressing heterogeneity from genetic and neural, to clinical units of analysis. Recent developments have also suggested the importance of going beyond the Diagnostic and Statistical Manual of Mental Disorders (DSM-5) defined categorical boundaries and attempting to identify homogenous clinical subgroups across individuals.

From these statements derives the inspiring idea of creating the present Research Topic, in order to give a collection of contributions under the frame of “heterogeneity,” “specificity” and “comorbidity” at clinical and neurobiological levels toward a better understanding of etiology and treatment. Indeed, the scope of the Research Topic has been to shed light on ASD, either at the clinical or neurobiological level, keeping in mind the frame of NDDs. This multi-disciplinary effort aimed at bringing together contributions ranging from basic science to sophisticated latent variable modeling approaches to facilitate clarification of distinct aspects of clinical phenotype, thus paving the way to clinical translatability.

The Research Topic has been taken into consideration by several research groups and finally consists of 20 papers (2 review papers and 18 original studies). In this editorial, we will discuss different themes that we have identified across the contributions: (1) Genetic variations and biological implications; (2) Neuroimaging and electrophysiological application; and (3) Characterization of the clinical phenotype. We conclude with a discussion on future directions in the field.

## Genetic variations and biological implications

As far as genetic variations and biological implications, it can be asserted that “synaptopathies”, involve disruption to genes expressed at the synapse and account for between 0.5 and 2% of autism cases. The Phelan McDermid Syndrome (PMS, also known as 22q13 deletion syndrome) and NRXN1 deletions (NRXN1ds) are two synaptopathies associated with autism. PMS often incorporates disruption to the SHANK3 gene, implicated in excitatory postsynaptic scaffolding, whereas the NRXN1 gene encodes neurexin-1, both implicated in trans-synaptic signaling in the brain.

Cooke et al. describe the Synaptic Gene (SynaG) study from the AIMS2-TRIALS project, which adopts a gene-first approach and comprehensively assesses these two syndromic forms of autism.

In the second paper focused on PMS, Isenstein et al., reported a habituation electrophysiological study (EEG-ERP) on PMS subjects to understand hyporesponsiveness in this clinical population. This study suggests that while neural response and habituation are generally preserved in PMS, genotypic and phenotypic characteristics may drive some variability.

Autism is often associated with potential risk factors that may alter the expression of certain receptors; for example, Liu et al., in a study of *in vitro* human neural progenitor cells, demonstrated that maternal diabetes may be correlated with the onset of ASD. Indeed, they showed that hyperglycemia, due to maternal diabetes, induces suppression of oxytocin receptors that contributes to social deficits in offspring, through a process of oxidative stress and epigenetic methylation.

Along a similar line of research Mariggio et al. focused on the genetic expression of the dopaminergic system (DS), possibly involved in the pathophysiology of ASD and attention-deficit/hyperactivity disorder (ADHD). Specifically, single nucleotide polymorphisms (SNPs) DRD1 and DRD2 dopamine receptors might be considered as potential risk factors for ASD and ADHD, thus being only DRD2-12 (rs7131465) significantly associated with a higher risk for the ASD/ADHD overlap.

Among biological risk factors also potential involvement of the immune system in the etiopathogenesis of ASD has been investigated. Specifically, De Giacomo et al. investigate the levels of immunological markers in peripheral blood of children with ASD founding that regulatory B cells and T cells were decreased in ASD subjects having a possible role in ASD pathophysiology.

## Neuroimaging and electrophysiological application

In this Research Topic neuroimaging and electrophysiological applications have been particularly used to study the effects of treatment and to understand how alterations in the brain correlate with clinical aspects of the autistic phenotype.

Both functional and structural MRI methodologies are represented in the studies included. Lan, Xu, Yu et al., based on resting-state data, reveal a possible relationship between atypical visual attention and poor learning ability in subjects with ASD, while the fMRI entropy connectivity method used by Yu et al. reveals that a combination of abnormal top-down and bottom-up information processing accelerates the deoptimization of brain networks and potentially affects cognitive activities in patients with ASD. Lan, Xu, Wu et al. reported spontaneous activity changes in terms of fMRI acquisition, in the visual and language-related brain regions in the ASD population, while the correlation with multiple clinical indexes did not appear significant.

Though in smaller sample sizes cohorts, preliminary interesting results are reported in structural MRI studies, especially in terms of structure-function connections. A correlation with clinical indexes (in terms of ADOS and ADI-R scores) has been reported in Lucibello et al., where cortical thickness and gyrification alterations in preschoolers with ASD are reported. In a longitudinal cohort of subjects at risk for autism, Godel et al. detected a regional structural MRI index of gray-white matter contrast (GWC), founding that early onset of ASD symptoms (i.e., prior to 18 months) was specifically associated with slower GWC rates of change during the second year of life, in areas related to the central executive network. In the paper from Chen et al., significant differences between the ASD group and the TD group in surface area, cortical volume, and cortical thickness were found and correlated with language, considering two ASD subgroups according to their response to speech ABR results, were detected, too.

Interestingly, two papers report (Sun et al.; Yu et al.) the application of neuroimaging/electrophysiology respectively serving as treatment monitoring or rehab intervention. In particular, Yu et al. reported a correlation between the reduction of socio-communicative symptoms and Default Mode Network activity in 3–6 years children with ASD after a 12-week mini-basketball training program. Another article in our Research Topic, by Sun et al., proposed a trial through tDCS stimulation in the dorsolateral prefrontal cortex area of children with ASD (4–12 years) and analyzed EEG mismatch negativity response in pre and post-treatment; even though a trend was detected, they did not found significant differences between ASD and healthy subjects, while a meaningful correlation of mismatch negativity response and symptomatology was detected.

The functional infrared-spectroscopy (fNIRS) technique has been recently reported as an efficient tool to investigate brain activity in the autism field (Zhang and Roeyers, 2019); in this Research Topic, Conti et al. systematically reviewed 13 papers applying fNIRS focused on young subjects (preschoolers) with ASD or infants at high risk of developing ASD, either in resting-state or task-evoked conditions. Findings confirm that the fNIRS application can represent a promising tool for potentially detecting autism traits, even in this very young population.

## Characterization of ASD clinical phenotype

Four articles in this Research Topic are focused on the characterization of ASD phenotype and related clinical implications. In particular, Operto et al., compared adaptive skills, emotional/behavioral problems, and parental stress among children with different severity levels of ASD symptoms, reporting a strong role of adaptive behavior in modulating the presence of internalizing problems, thus suggesting the importance of taking it into account in the rehabilitation program and family support.

Beyond emotional/behavioral problems, DSM5 (APA, 2013) introduced the possibility of double diagnosing ASD and ADHD conditions, and existing literature suggests shared neurobiology between the two conditions (Di Martino et al., 2013). Interestingly, Aiello et al., tried to disentangle the clinical phenotype and specificity of the two co-occurring conditions in relation to autism traits [C-AQ-(Auyeung et al., 2008)] and empathy [C-EQ (Auyeung et al., 2009)], by comparing children with ASD with and without comorbid ADHD with children presenting ADHD only and children with typical development. The reliability of the C-AQ and C-EQ as behavioral markers to differentiate ASD (regardless of comorbid ADHD) from an ADHD condition and TD was confirmed.

Considering the dimensional approach to the autism spectrum, it is of interest the detection of autistic traits in the general population, too. In this frame, Vaiouli and Panayiotou used regression models to establish cross-sectional associations between autistic traits, alexithymia, and social-emotional difficulties in 275 young adults (e.g., college students), thus providing evidence of the influence of different alexithymic facets on the relationship between autistic traits and social-emotional challenges in young adults.

Research on ASD parents is another issue of great interest in the field, thus often representing a direct window on the broader autism phenotype (Sucksmith et al., 2013) and related shared neurobiological bases, too (Billeci et al., 2016). In the paper from Uljarevic et al., the relationship between social motivation in children with ASD and their parents was investigated, through the administration of the Social Responsiveness Scale (SRS). The study established that low social motivation in children with ASD may be driven, in part, by lower social motivation in one or both parents.

First-degree relatives of individuals with ASD may show mild deficits in cognitive flexibility as reported by Cheng et al. Indeed, the authors investigated first-degree relatives of individuals with ASD, either at the clinical level in terms of BAPQ (Piven et al., 1997) and CFI (Dennis and Vander Wal, 2010) or at the neuroeletrophysiological level (ERP), reporting cognitive flexibility deficits at both levels in ASD parents. The cognitive flexibility difficulties were related to autistic traits, thus representing a neurocognitive endophenotype of ASD.

## Conclusion and future directions

The published papers on this Research Topic highlight the complexity of performing research in the field of ASD because of neurobiological heterogeneity and phenotypic expression. To conclude, we would like to mention the review paper from Nordahl et al. that is entirely in line with the core intention of the Research Topic. As a matter of fact, the authors summarize the findings of the Autism Phenome Project (APP), a longitudinal multidisciplinary study since 2006, that now includes over 400 subjects from 2 to 19 years of age investigated from medical, behavioral, and neuroimaging perspectives, with the final aim to catch heterogeneity of autism. This approach represents an effective model to investigate autism, showing the importance of translational contribution in developing better-tailored treatments and preventive strategies.

As suggested by Nordahl et al., the identification of subgroups is not meant to divide the autism community, but rather to improve individual care plans and redesign the care and social services for autistic people and their families.

All the published manuscripts emphasize future perspectives and ongoing challenges in the field. Hence, we would like to thank the contributors for their interesting and significant contributions and wish that this Research Topic stimulates further research potentially impacting the autistic community.

## Author contributions

All authors listed have made a substantial, direct, and intellectual contribution to the work and approved it for publication.

## Funding

This work was supported by the European Research Council (ERC) under the European Union's Horizon 2020 Research and Innovation Program (Grant No. 801715) (PUPILTRAITS) and (Grant No. 832813) (GenPercept) to MT and by AIMS-2-TRIALS network to EC.

## Conflict of interest

The authors declare that the research was conducted in the absence of any commercial or financial relationships that could be construed as a potential conflict of interest.

## Publisher's note

All claims expressed in this article are solely those of the authors and do not necessarily represent those of their affiliated organizations, or those of the publisher, the editors and the reviewers. Any product that may be evaluated in this article, or claim that may be made by its manufacturer, is not guaranteed or endorsed by the publisher.
